# Roles of Histone Deacetylases in Acute Myeloid Leukemia With Fusion Proteins

**DOI:** 10.3389/fonc.2021.741746

**Published:** 2021-09-01

**Authors:** Juan Zhang, Xuefeng Gao, Li Yu

**Affiliations:** Department of Hematology and Oncology, International Cancer Center, Shenzhen Key Laboratory of Precision Medicine for Hematological Malignancies, Shenzhen University General Hospital, Shenzhen University Clinical Medical Academy, Shenzhen University Health Science Center, Shenzhen, China

**Keywords:** HDACs, AML, leukemogenesis, epigenetic modification, oncogenic fusion protein, chromosomal translocation

## Abstract

Accurate orchestration of gene expression is critical for the process of normal hematopoiesis, and dysregulation is closely associated with leukemogenesis. Epigenetic aberration is one of the major causes contributing to acute myeloid leukemia (AML), where chromosomal rearrangements are frequently found. Increasing evidences have shown the pivotal roles of histone deacetylases (HDACs) in chromatin remodeling, which are involved in stemness maintenance, cell fate determination, proliferation and differentiation, *via* mastering the transcriptional switch of key genes. In abnormal, these functions can be bloomed to elicit carcinogenesis. Presently, HDAC family members are appealing targets for drug exploration, many of which have been deployed to the AML treatment. As the majority of AML events are associated with chromosomal translocation resulting in oncogenic fusion proteins, it is valuable to comprehensively understand the mutual interactions between HDACs and oncogenic proteins. Therefore, we reviewed the process of leukemogenesis and roles of HDAC members acting in this progress, providing an insight for the target anchoring, investigation of hyperacetylated-agents, and how the current knowledge could be applied in AML treatment.

## Introduction

Acute myeloid leukemia (AML) is characterized by genetic mutations and epigenetic alterations, marked by uncontrollable proliferation, blocked differentiation, and anti-apoptosis (1–3). And the majority of AML events are correlative with abnormal chromosomal translocations, which generates the oncogenic fusion genes. Mounting studies have demonstrated the central roles of fusion genes in initiating the leukemogenesis (4–6). And the successful strategies are paralleled by the decrease or degradation of chimeric proteins (7, 8). Commonly, the fusion partner in chimeric protein acts as a transcriptional protein interacting with the recruited corepressor complexes, which alters the expression of target genes that maintain the homeostasis of myeloid development, conferring the foundation of leukemic transformation (9, 10). Thereby, master the potential elements interacting with the fusion proteins is the prerequisite for targeting such oncogenic chimera.

Epigenetic modification has been acknowledged to paly crucial roles in the oncogenic transforming including AML (11, 12). Generally, epigenetic modification is not dedicated to some specific genes but serving for a vital regulator of transcriptional factors, which hold the specific capacity of DNA binding, whereby determining the potential transcriptional outcome (13–15). Thereby, the function of epigenetic modification is closely related to the cell-specific situation where the transcription factors are involved.

Accumulating evidences have been presented that epigenetic aberration prominently contribute to the leukemogenesis (16–18). As one of the major epigenetic regulators, histone deacetylases (HDACs) are indispensable in gene transcription. Dysregulation of HDACs has long been recognized as a crucial driver to hematological malignancies from initiation to metastasis, because they determine the fate of tumor cells, directing the cell to proliferate, differentiate, or be quiescent (13, 19). Therefore, the orchestration of HDACs is closely related to the cell development of both normal cells and tumor cells.

As acetyl group removers, HDACs control the accessibility of chromatin for transcription factors through switching the acetylated status, which finely tunes the transcriptional level of transcription factors and epigenetic modifiers, involving in development, cellular homeostasis, and carcinogenesis (20–22). And deregulated HDACs are associated with cell differentiation arrest, cell cycle disruption, DNA damage, and cell death (13, 23). Targeting the dysfunctional deacetylation in AML provides a promising strategy benefit for tumor treatment (24, 25). And experimental and clinical functions of HDAC inhibitors have been described by a number of reports (26–31), but the detailed mechanism acted by HDACs has not been elaborated. Comprehensively harness the roles of HDAC family members acting in leukemogenesis will provide us more precise prevision against such malignancy.

AML is frequently associated with chromatin rearrangement, including translocation and inversion, which generate oncogenic fusion proteins, among of which four most common chimeric proteins should be paid more attention, including AML1-ETO, PML-RARα, CBFβ-MYH11, and MLL-MLLT3 (4, 6, 32–34). Here we attempt to summarize the mutual interactions between HDACs and oncogenic fusion proteins involved in AML, providing a reference for the precise application of HDAC inhibitors and novel drug exploration against AML.

## Acute Myeloid Leukemogenesis and Classification

**Figure 1:**
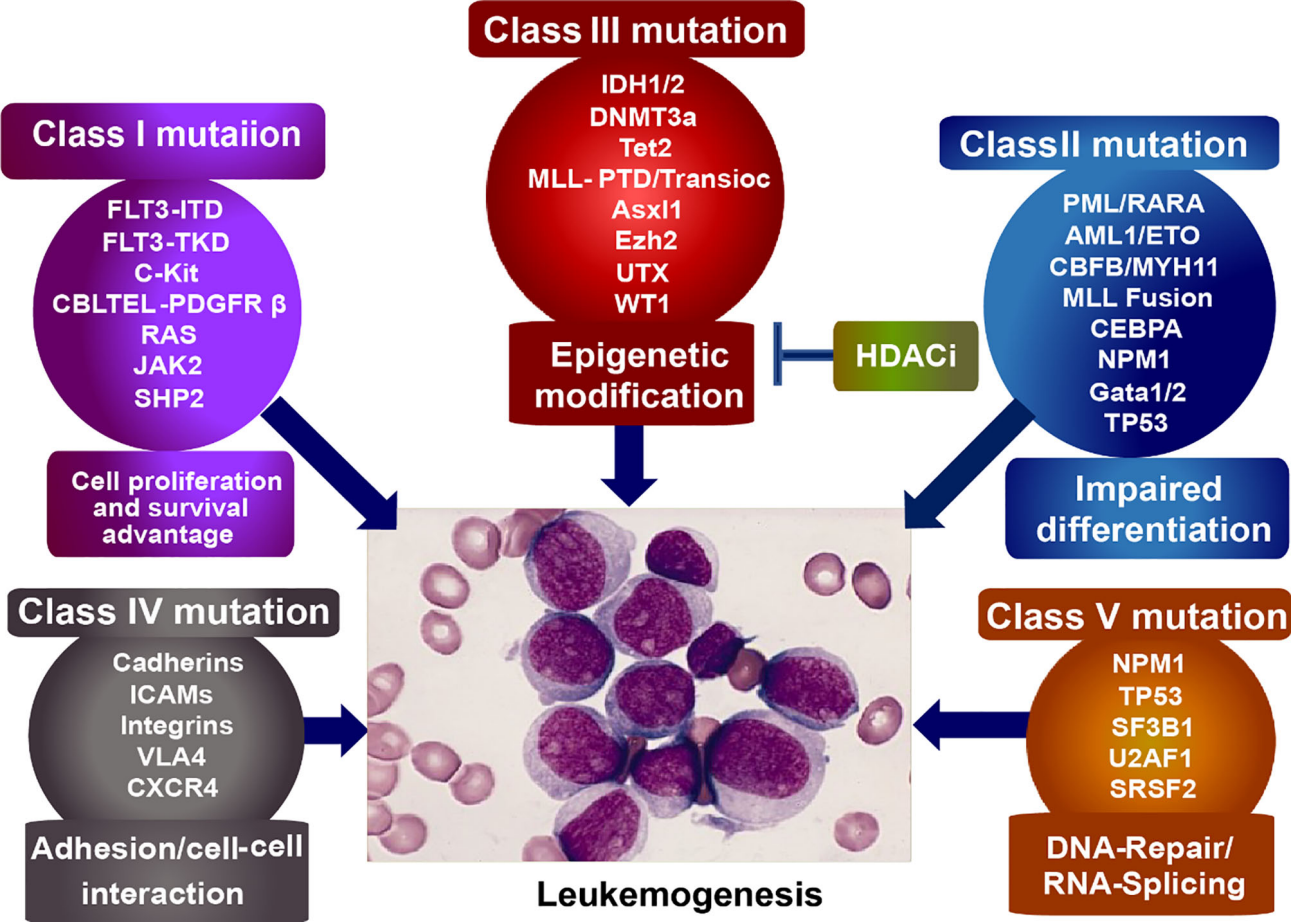
Classification of mutagenic genes eliciting leukemogenesis. Class I mutation, provides tumor cells with survival/proliferation advantages; Class II mutation, disturbs the cell differentiation; Class III mutation, epigenetically dysregulates the tumor suppressor/activator; Class IV mutation, alters cell adhesion and cell-cell interaction, leading to the flexible motility and migration. Class V mutation, dysregulates DNA-repair and RNA-splicing.

Acute myeloid leukemogenesis is a complicated progress involved in genetic and epigenetic alterations, leading to uncontrolled proliferation, arrested differentiation, and myeloid dysfunction (1, 2). And the altered genes can be subdivided into five categories (**Figure 1**): Class I mutations, activators of tyrosine kinase, such as *c-Kit, Flt3*, and *BCR-ABL*, provide the hematopoietic progenitors with survival/proliferation advantage. Class II mutations, transcriptional factors such as *NPM1, CEBPA*, and *TP53* as well as oncogenic fusion genes (e. g. *AML1-ETO*, *PML-RARα*, and *CBFβ-MYH11*), arrest the differentiation of hematopoietic cells. Mutations emerging in either class I or class II do not result in leukemogenesis until the both happen to mutate. When differentiation of hematopoietic cells is hindered by Class II mutations, Class I mutations would autonomously proliferate, initiating the leukemogenesis. Class III mutations, epigenetic regulatory molecules (e. g. TET2, IDH1 and IDH2, DNMT3A, and HDACs), silence/activate the tumor suppressor genes/pro-tumor genes. And the class IV mutations involve genes that alter cell adhesion and cell-cell interaction, leading to the flexible motility and migration. Class V mutation includes genes dysregulating DNA-repair (e.g.TP53 and NPM1) and RNA-splicing (35–40). We focus on the epigenetic abnormalities of histone modification in the progression of leukemogenesis.

The subtypes of AML are majorly classified by two systems: French-American British (FAB) classification used earlier, and World Health Organization (WHO) classification, which has replaced the former (2). According to FAB classification (**Table 1**), AML can be grouped into eight subtypes from M0 to M7 based on the leukemic cell development and maturity. Among of them, M0 to M5 derived from the progenitors of white blood cells; M6 start with early forms of red blood cells; and M7 originates in the early forms of platelets (41–44).

**Table 1:** FAB subtype of AML.

FAB subtype	Stage of cell development	Percentage of adult AML patients	Prognostic stratification
0	AML with undifferentiated myeloblasts	5%	Worse
M1	AML with minimal maturation	15%	Average
M2	AML with maturation	25%	Better
M3	Acute promyelocytic leukemia (APL)	10%	Best
M4	Acute myelomonocytic leukemia	20%	Average
M4 eos	Acute myelomonocytic leukemia with eosinophilia	5%	Better
M5	Acute monocytic leukemia	10%	Average
M6	Acute erythroid leukemia	5%	Worse
M7	Acute megakaryoblastic leukemia	5%	Worse

According to WHO classification (45–50), AML is subdivided into 6 categories (**Table 2**): 1) AML with recurrent genetic abnormalities, involving in translocation, inversion, deletion, and mutation; 2) AML with myelodysplasia-related changes (MRC), a kind of multilineage dysplasia; 3) therapy-related myeloid neoplasms (t-MN), such as chemotherapy and radiation; 4) AML, not otherwise specified (NOS), including M0, 1, 2, 4, 5, 6, 7, acute basophilic leukemia, and acute panmyelosis with fibrosis; 5) myeloid sarcoma; 6) myeloid proliferations related to Down syndrome (DS). AML with recurrent genetic abnormalities contains balanced translocation/inversion, and mutation. The balanced translocations include t (8,21) (q22;q22.1) (AML1-ETO); inv (16) (p13.1q22)(CBFβ-MYH11); t (9,11)(p21.3;q23.3)(PML-RARα); t (6,9) (p23;q34.1) (KMT2A-MLLT3); inv (3)(q21.3q26.2)(DEK-NUP214); t (1,22)(p13.3; q13.1) (Gata2, Mecom); Rbm15-MKL1, and Bcr-Abl1. Here we will discuss the four most common fusion proteins involved in AML, focusing on the roles of HDACs functioning in the fusion proteins.

**Table 2:** WHO classification of AML.

WHO classification of Acute myeloid leukemia (AML)				
AML-associated oncofusion proteins				
Chromosomal translocation	Oncofusion protein	Frequency of occurrence	Prognosis	FAB
t (8,21)(q22;q22)	AML1-ETO	10-15%	Favorable	M2
t (15,17)(q22;q21)	PML-RARαβ	6-15%	Favorable	M3
inv (16)(p13q22)	CBFb-MYH11	3-10%	Favorable	M4
der(11q23)	MLL-fusions	5-8%	Variable	M4/M5
t (9,22)(q34;q11)	BCR-ABL1	1-2%	Adverse	M1/M2
t (6,9)(p22;q34)	DEK-NUP214	<1	Adverse	M2/M4
t (1,22)(p13;q13)	RBM15-MKL1	<1	Intermediate	M7
t (8,16)(p11;p13)	MYST3-CREBBP	<1	Adverse	M4/M5
t (7,11)(p15;p15)	NUP98 -HOXA9	<1	Intermediate	M2/M4
t (12,22)(p12;q11)	MN1-TEL	<1	Variable	M4/M7
inv (3)(q21;q26)	RPN1-EVI1	<1	Adverse	M1/M2/M4/M6/M7
t (16,21)(p11;q22)	FUS -ERG	<1	Adverse	M1/M2/M4/M5/M7
**AML with mutations**				
NPM1; CEBPA (biallelic mutation); RUNX1; myelodysplasia-related changes; Therapy-related myeloid neoplasms				
**AML, not otherwise specified (NOS)**				
Undifferentiation; Minimal maturation; Maturation; Acute myelomonocytic leukemia; Acute monoblastic and monocytic leukemia; Pure erythroid leukemia; Acute megakaryoblastic leukemia; Acute basophilic leukemia; Acute panmyelosis with myelofibrosis; Myeloid sarcoma				
**Myeloid proliferations associated with Down syndrome**				
**Transient abnormal myelopoiesis (TAM) associated with Down syndrome**				
**Myeloid leukemia associated with Down syndrome**				

## HDACs Classification and Functions

Nucleosome, constituting the fundamental units of chromatin, is an octamer polymerized by four types of histones (H2A, H2B, H3, and H4), wrapped by 146 base-pair DNA. Each histone contains a structural domain and an unstructured tail of 25-40 amino acid residuals, which can be altered *via* post-translational modification, including acetylation, methylation, phosphorylation, and ubiquitination (51, 52). And the modification of histone residuals will determine the chromatin accessibility to transcription factors, keeping them activated or silent. Thereinto, the homeostasis of acetylation generally depends on the dynamic regulation of histone deacetylases (HDAC) and histone acetyltransferases (HAT) (53, 54).

**Figure 2:**
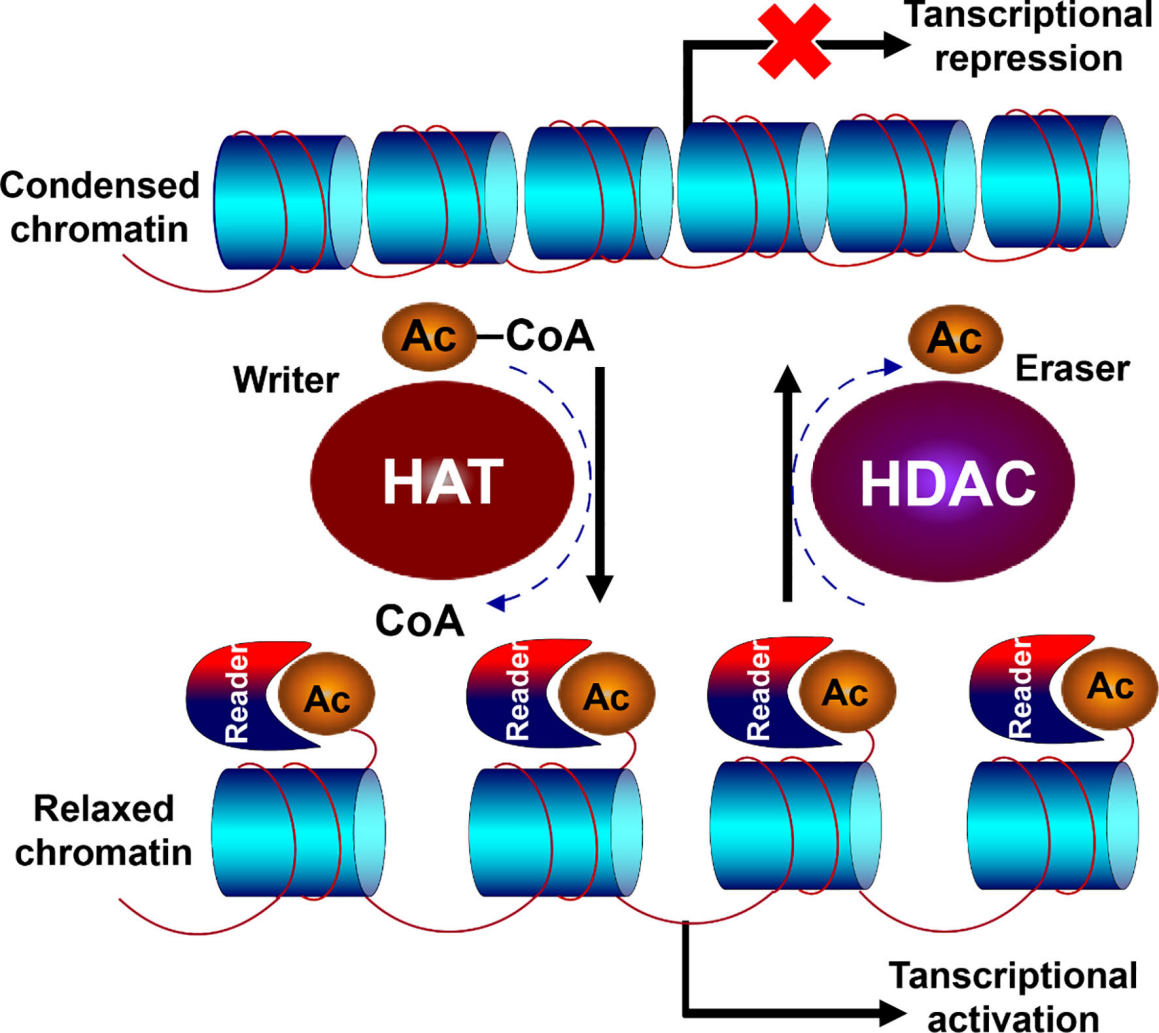
Opposite function of HDACs and HATs. HDAC and HAT play opposite roles in the epigenetic modification of chromatin. HAT transfers the acetyl group from acetyl coenzyme A to lysine residual of histone N-terminal with positive charge, which binds to DNA strand with negative charge and prevents the chromatin from being condensed, allowing the chromatin relaxed for gene transcription. Oppositely, HDACs remove the acetyl group from histone tail, and subsequently condenses the chromatin, resulting in transcriptional inhibition.

HDAC and HAT play opposite roles in the epigenetic modification of chromatin, especially the histone proteins, where HATs allow the chromatin relaxed for gene transcription, and HDACs condense the chromatin making it inaccessible for transcriptional factors (**Figure 2**). HAT transfers the acetyl group from acetyl coenzyme A to lysine residual of histone N-terminal with positive charge, which binds to DNA strand with negative charge and prevents the chromatin from being condensed, thereby keeping the chromatin loosened available for the binding of transcription factors with DNA. Oppositely, HDACs favor to compact the chromatin, preventing the gene transcription. They remove the acetyl group from histone tail, and subsequently condense the chromatin, resulting in transcriptional inhibition (55–57). Therefore, the dysregulation is inevitable when the balance is disrupted between HDACs and HATs.

**Figure 3:**
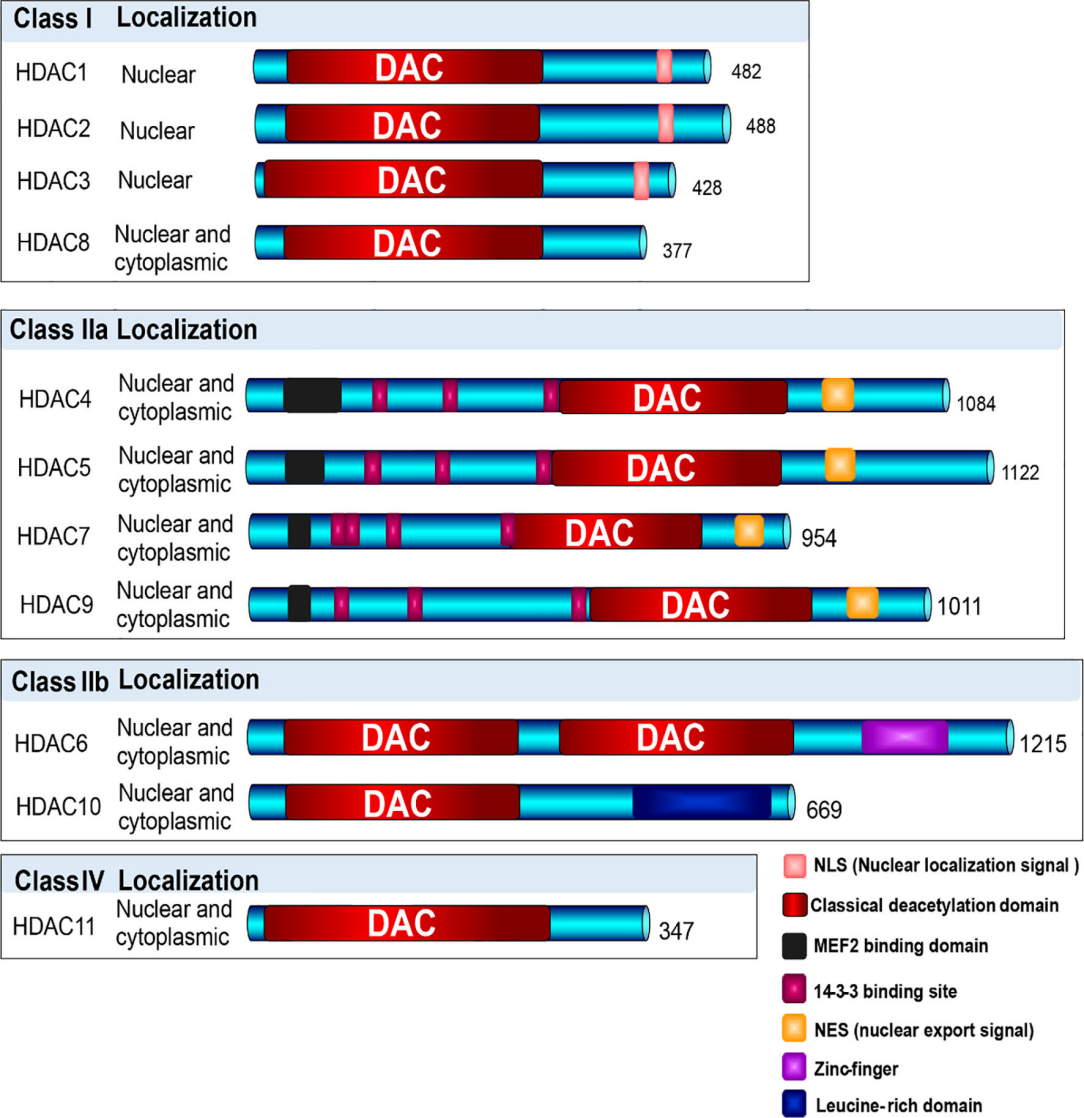
Classification of HDACs. Based on the phylogenetic analysis, sequence homology to yeast protein, and domain organization, HDAC enzymes are categorized into four families (class I, IIa, IIb, III and IV). Three of them contain the Zn^2+^ dependent catalytic domain, which are referred to as classical HDACs, and class III members are NAD^+^-dependent, which were not involved in this description. Class I HDACs contains HDAC1, 2, 3, and 8, which majorly localize in nuclear; Class II HDACs include Class IIa (HDAC4, 5, 7, 9) and Class IIb (HDAC6, 10), which shuttle between nuclear and cytoplasm; and Class IV contains only HDAC11, shuttling between nuclear and cytoplasm.

HDACs are universally spread in eukaryotes, which belong to a superfamily composed of 18 proteins with conserved deacetylase domain (21, 23). Based on the phylogenetic analysis, sequence homology to yeast protein, and domain organization, these proteins can be categorized into four families (class I, IIa, IIb, III and IV) (**Figure 3**). Three of them contain the Zn^2+^ dependent catalytic domain, which are referred to as classical HDACs, and class III members are NAD^+^-dependent, called sirtuins, which possesses deacetylase activity but is unrelated to HDACs, and will not be involved here. Distinguished by structure, enzymatic function, and localization, they display similar and specific functions during the regulation of gene expression (13, 21, 58).

Class I HDAC family is consist of HDAC1, 2, 3, and 8, which are homologous to yeast protein reduced potassium dependency-3 (Rpd3) (21, 59). They are chiefly expressed in nuclear, consisted by classic deacetylase domain, nuclear localization signal, showing high enzymatic activity to their substrates. Approximately 400 amino acids consist of each member, the catalytic domain contains two histidine residues, two aspartic acid residues and one tyrosine residue with Zn^2+^. And they generally function as gene transcriptional repressors. For instance, HDAC1 and HDAC2 bear closely identical structure and similar function, and usually work together in the repressive complexes, such as corepressor for element-1-silencing transcription factor (CoREST), nucleosome remodeling and deacetylase (NuRD), and transcription regulator family member switch-independent 3 (Sin3) complexes. HDAC3 generally emerges in another type of repressive complexes, such as N-CoR–SMRT complex. HDAC8 has been described to cooperate with SMAD3/4 complex, promoting the cell proliferation and migration (60–65).

According to the number of catalytic domains, Class II HDAC family can be subdivided into Class IIa (HDAC4, 5,7, 9) and Class IIb (HDAC6, 10), which can shuttle between nucleus and cytoplasm (66, 67). Class IIa HDAC members are grouped by a functionally important N-terminal domain, which mediates DNA-binding and nuclear-cytoplasmic shuttling. HDAC trafficking is regulated by nuclear export signal (NES) and binding sites for14-3-3 proteins. Upon 14-3-3 protein binding, cytoplasmatic retention or nuclear export of class IIa HDACs will be stimulated depending on the phosphorylation of 14-3-3 binding sites, which can be regulated by protein kinase-D, Ca^2+^/calmodulin-dependent kinases (CaMKs), and checkpoint kinase-1 (CHK1). And subsequently the transcriptional repressors will be regulated *via* binding with myocyte enhancer factor 2 (MEF2) binding domain, conferring signal responsiveness to downstream genes. When bound with Class IIa HDACs, MEF2 makes them a transcriptional repressor, whereas bound with HATs p300, MEF2 then converted them into a transcriptional activator. And the deregulated balance of HDAC and HAT will subsequently lead to diseases (68–71). Class IIb HDACs are atypical ones. HDAC6 contains two deacetylase domains and a C-terminal zinc-finger, which functions as a major cytoplasmic deacetylase targeting alpha-tubulin and HSP90, regulating cell motility, adhesion, and chaperone function (72, 73). Besides, binding with ubiquitin *via* zinc finger domain HDAC6 can regulates the aggresome formation, autophagy, heat shock factor-1 (HSF-1), and function of platelet derived growth factor (PDGF) (74, 75). HDAC10 holds single deacetylation domain and a leucine-rich domain. It possesses properties of immunoregulator, against the tolerogenic molecule PD-L1, implying an epigenetic target for immunotherapy. Overexpression of HDAC10 has been demonstrated to accelerate the progress of carcinogenesis. Deletion of HDAC10 in antigen-presenting cells (APCs) can increase the expression of MHC class II molecules and repress the transcription of PD-L1, which is associated with enhancement of immune system (76–79).

HDAC11, as the sole member of Class IV HDAC family, structurally similar to class I and II, mainly distributes in nucleus and acts as a repressor of IL-10 (80). It can regulate the dynamic balance between immune activation and tolerance. Upregulation of HDAC11 has been shown in various cancer cells (81, 82).

Besides, an increasing number of non-histone proteins have been identified as substrates of HDACs, such as p53, Stat3, Hsp90, GATA1, Tubulin, and β-catenin, which display vital roles during the progress of carcinogenesis (83–85). *Via* deacetylation, HDAC1 can affect the stability of tumor suppressor gene p53, arresting the interaction with DNA, inverting the function of p53. HDAC1 can also directly lead to the deacetylation of GATA1, repressing the gene transcription. HDAC6 is associated with the modulation of Akt and Stat3 signaling *via* regulation of Hsp90 acetylation in multiple myeloma cells. Deletion of HDAC6 will result in reducing phosphorylation of Stat3, which results in related genes inactivation (22, 86, 87).

**Figure 4:**
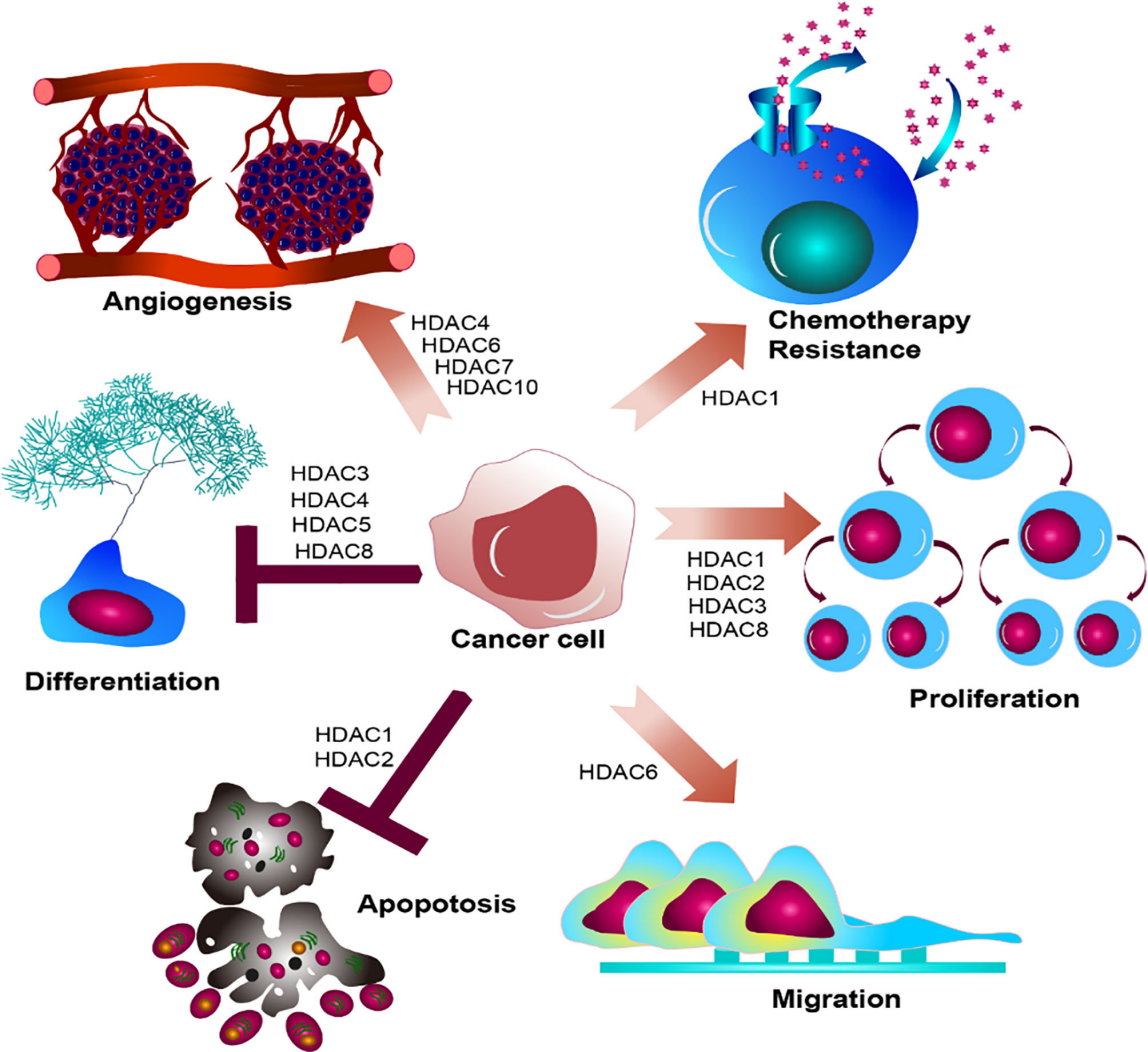
Summary of HDACs functioning in cancer cell. HDAC family members determine biological effect of oncogenic hallmarks emerging in cancer cell, disrupting the regular cell development in cancer cell, leading to dysregulated differentiation, proliferation, migration, chemotherapy resistance, and angiogenesis.

Taken together, HDACs participate in the regulation of key transcriptional factors involving in the gene transcription, cell apoptosis, cell cycles, and signal transduction, which depicts the pivotal roles of HDACs functioning in epigenetic modification and gene transcription. The histone modification determines the accessibility of chromatin, which will make genes activated or silent. Inevitably, dysregulated histone modification will lead to dysfunctional cell development, which is strongly associated with carcinogenesis. Disruption of specific HDACs usually associates with dysregulation of differentiation, proliferation, migration, chemotherapy resistance, and angiogenesis (**Figure 4**). Overexpression of HDAC usually emerges accompanying with leukemogenesis and the other tumor. They act to close the nucleosomes, inhibiting the expression of tumor suppressor genes. HDAC inhibitor, as an agonist of HDAC, can alter the abnormal hypoacetylation level of histone, and subsequently elicits cell differentiation and apoptosis, demonstrating the indispensable roles of HDACs in tumorigenesis (88–91). Harnessing the function of HDACs is the premise indicating to precisely target the master alterations.

## HDACs in Leukemogenesis

Although HDAC mutations in AML are relatively rare compared to solid tumors, HDAC proteins are abnormally recruited to oncogenic fusion proteins, such as AML1-ETO, CBFB-MYTH11, PML-RARα, and MLL-fusions, which function as vital roles in onsetting and promoting the progress of leukemogenesis (4, 13, 31). And HDAC inhibitors, as a series of compounds that neutralize the activities of HDACs, have long been utilized in treatment of AML for pre-clinical studies, which have some extend shown beneficial outcomes (26, 27, 30, 31). And the multiple functions of HDAC inhibitors have been discussed in numerous research articles and reviews (which will thereby not be included in this review). However, the mutual interaction between HDACs and AML has not been comprehensively described. And we choose the most frequent events of chromosomal translocation emerging in AML to elucidate the reciprocal functions of AML and HDACs.

## HDACs in AML With AML1-ETO

One of the well-studied AML subtypes is t (8, 21) AML, which occurs in approximately 10-15% of total AML cases, and 18-40% of M2 AML (92–95). The translocation is generated by the fusion of AML1 gene (Runx1) on 21q22.1 and ETO gene (Runx1T1) on 8q22, leading to the forming of AML1-ETO fusion protein (5, 34, 96). It can invert the original function of AML1, performing opposite function during the leukemogenesis. The fusion protein AML1-ETO provides the DNA-binding domain *via* the hematopoietic master regulator AML1 and transcriptional domain *via* ETO, targeting the AML1 target genes. It substitutes the original function of AML1 and disrupts cellular processes involved in the myeloid proliferation, differentiation, and genome stability (95, 97, 98).

**Figure 5:**
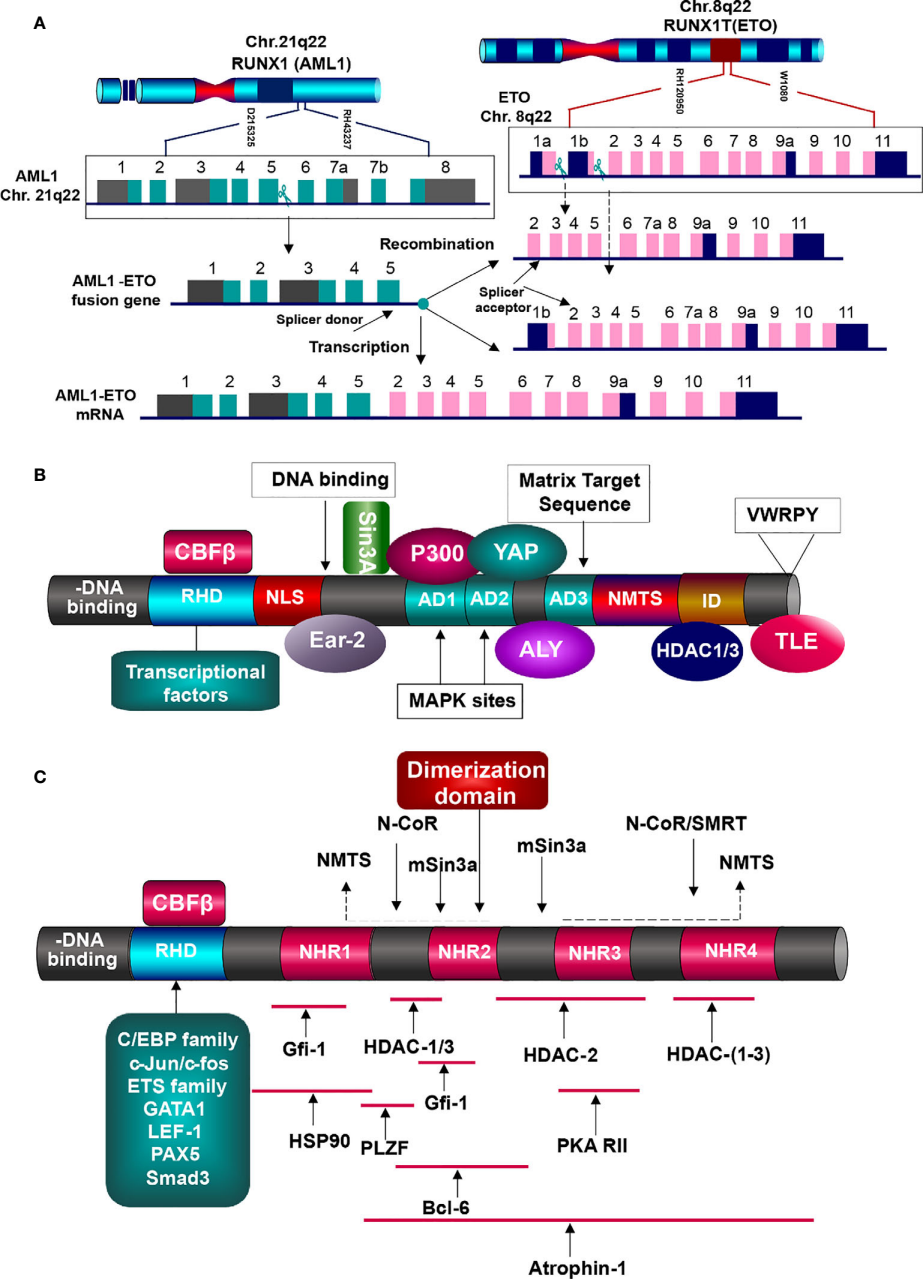
Generation and function of AML1-ETO fusion protein. **(A)** Generation of AML1-ETO. ETO gene on Chr.8q22 is consist of 13 exons, containing two breakpoints, but one splicer acceptor in exon2. AML1 gene is made up of nine exons with one breakpoint and one splicer donor. Absence of splicer acceptor in exon1b, the two genes generate the only fusion mRNA. **(B)** AML1 protein structures and partner proteins. AML1 is composed of DNA-binding domain (RHD) and other domains related to signal transduction, transcriptional factors binding, epigenetic modifiers interaction, and TLE co-repression, which can interact with HATs (MOZ, CBP, and p300) and HDACs (HDAC1, 2, and 3), resulting in gene activation or inhibition. HATs, histone acetyltransferases; HDACs, histone deaccetylases; RHD, runt homology domain; NLS, nuclear localization signal; AD, activation domain; NMTS, nuclear matrix targeting signal; ID, inhibitory signal; VWRPY sequence. **(C)** AML1-ETO fusion protein and the interacting partners. AML1 contributes the DNA-binding domain RHD, which binds with various transcription factors, but lacks of domains to elicit regular functions. And nearly whole of ETO structure is involved in the fusion, including the four NHRs. They interat with corepressive complexes, HDACs, and other molecules, initiating the oncogenesis.

To understand the mutual interactions between AML1-ETO and HDACs in detail, we firstly figure down the functions of AML1 and ETO in normal condition and AML1-ETO in tumorigenic environment. AML1 functions as a master organizer, which in charge of regulating the hematopoietic specific promoters and enhancers. It widely spreads in hematopoietic system, cooperating with multiple lineage-specific transcriptional regulators, such as the driving of endothelial hematopoietic transition (99, 100). AML1 gene on 21q22 is composed of nine exons, with three breakpoint cluster regions (BCR) in intron5. The structure of AML1 is composed of conserved runt homology DNA binding domain (RHD), activation domain (AD), nuclear matrix-targeting signal (NMTS), proline-rich domain (PY), two inhibitory domains (ID), and an additional C-terminal motif with five amino acid (VWRPY), working as a recognition and recruitment signal for Groucho/TLE family. Besides, it contains two promoters: distal promoter P1 and proximal promoter P2. Both promoters include the AML1-binding sties, which can be regulated by itself and other AML1 transcriptional factors. RHD is in charge of recognizing and binding to DNA sequences, and localizing the AML1 transcriptional factors in nucleus. It also contributes to the binding of core biding factor β (CBFβ), which does not interact with DNA, but increases the α subunit affinity to DNA binding and stabilizes the complex (**Figure 5A**) (101–103).

AML1 functioning as an activator or repressor is determined by its interaction with corresponding transcriptional factors and co-factors, rather than itself features (95, 104, 105). It has been shown to interact with various chromatin modifiers and remodelers (**Figure 5B**). For instance, its activation can be stimulated by binding with lysine acetyl transferase MOZ (MYST3), and the same to transcriptional co-activators P300 and CBP. They function as integrators, which bind with AML1 and other transcriptional activators driving the hematopoietic promoters (106). ALY expressed in nucleus can bind to the activation domain of AML1, forming multimers and bridging the interaction of AML1 and other transcription factors. c-Yes tyrosine kinase associated protein (YAP) binds to PPPY motif in the AML1 C-terminal activation domain, enhancing the activity of AML1 (105, 107, 108).

Furthermore, AML1 may function as a repressor of HDAC complex (15). Researches have demonstrated that it can inhibit the transcription of p21 *via* binding the promoter of p21 with AML1 VWRPY Groucho/TLE interaction domain. Through binding with co-repressors such as Sin3A and Groucho/TLE, it recruits HDACs to repress the transcription (15). And the HDAC inhibitor Trichostatin A can impair such suppression, demonstrating that HDACs contribute to AML1-mediated inhibition. It is also associated with HDAC1, 2, 3 and histone H3 lysine 9 methyltransferase SUV39H1 (KMT1A), leading to transcriptional suppression. In myeloid cells, AML1 binding with CCAAT/enhancer binding protein alpha (C/EBPα) and PU.1 can activated the macrophage colony-stimulating factor receptor (M-CSFR) expression (104, 109).

Meanwhile, AML1 can also been inhibited by corresponding transcription factors. For instance, bound with forkhead box P3 (FOXP3), it can suppress the expression of interleukin2 (IL2) and interferon gamma (IFN-γ) in T regulatory cells. It is multifunctional in the regulation of hematopoiesis, including cell differentiation, proliferation, and apoptosis. And the aberration of AML1 will speculatively deregulate the normal cellular development, which is involved in carcinogenesis (15, 109).

Eight-twenty-one (ETO) gene on 8q22 is consist of 13 exons, with one BCR in intron1a and three BCRs in intron1b, which generate different variants but create the same fusion gene AML1-ETO, because it supplies only one splice acceptor in exon2, as the exon1b lacks of splice acceptor (104, 110). ETO protein possesses three proline-serine-threonine (PST)-rich regions and four conserved nervy homology regions (NHR), involved in neuronal development of Drosophila embryos. The PST-rich domains contain multiple potential kinase phosphorylation sites (SP and TP). NHR1 is homologous to the Drosophila TATA-box-associated factors, including TAF110. NHR2 domain, containing a hydrophobic amino-acid (a.a.) heptad repeat, plays crucial role in the oligomerization between ETO family members, forming homo-/hetero-dimerization. NHR3 is with predicted coiled-coil structure. NHR4 is homologous to myeloid-Nervy-DEAF1 (MYND) homology domain, with two predicted zinc-finger motifs, which is required for the protein-protein interaction. For instance, ETO is associated to the co-repressors mSin3 and nuclear receptor corepressor (N-CoR), thyroid hormone receptor (SMRT), as wells as HDAC1, 2, 3. Via binding with NHR4 DNA binding domain, it can interact with the co-repressors N-Cor, SMRT, and mSin3A, which will then tether the DNA-binding proteins to HDACs, resulting in repressive transcription (105, 111). Researches have shown that ETO and AML1-ETO can pull-down by HDAC activity *via* Co-IP, bearing out the repressive role of AML1-ETO through the recruitment of HDACs to AML1 target genes (112). Such function may instead of AML1 complex which originally worked as an activator involving in the histone acetyl transferases p300/CBP. It is similar to the leukemogenic mechanism of APL. Fusion proteins PML/PLZF-RARα increase the affinity of RARα to co-repressors and RARα target genes (113). It also interacts with Atrophin-1, chaperon heat-shock protein (HSP90), PLZF, Gfi-1, and Bcl-6, functioning as a corepressor of transcriptional factors. Via NHR1 and NHR2, ETO can binds to Gfi-1 and Gfi-1b, contributing to the recruitment of HDACs, which subsequently repress the activity of Gfi-1/Gfi-1b proteins (111, 112, 114). Depending on the DNA binding site provided by RHD of AML1, AML1-ETO may perform as a repressor or activator of the AML1 target genes (115).

In AML1-ETO fusion protein, the important features of AML1 are lost: 1) the c-terminal activation domain interacting with co-activators; 2) domains in charge of binding with co-repressors such as Sin3 and TLE; NLS domain functioning as nuclear matrix-targeting signal. And such lost will subsequently result in the dysregulation of hematopoiesis. The AML1-ETO fusion protein can affect the expression of both AML1 target genes and other related genes. As a part of AML1-ETO, AML1 recruits HDACs to the promoter, which suppress the expression of relative target genes. In normal, these target genes are required for regulating cell growth and preventing hemopoietic cells from transformation. And abnormally, the target genes are suppressed and lose their control, leading to cell overgrowth (107).

In t (8,21) AML, a number of genes critical to normal hematopoiesis are up-regulated by AML1, while AML1-ETO disrupts such trans-activation. AML1–ETO fusion protein recruits various transcriptional factors, epigenetic modifiers such as HDACs, PRMT1, and p300, forming the first aberration vital for the t (8,21) AML onset (13). And then it can collaborate with the secondary mutations including c-Kit, FLT3, and RAS. Via recruiting the HDAC1, 2, 3, AML1-ETO can silence the target genes and block the cell differentiation and transformation (95, 116). AML1 contributes the DNA binding domain RHD to a number of transcriptional factors (such as Ets-1, LEF-1, C/EBPα, PU.1, MEF, Pax5, and GATA1) and epigenetic modifiers, but defaults the subsequential elements for activation, which are replaced by nearly entire ETO. The well-known binding protein of AML1 is CBFβ, which efficiently binds to RHD of AML1 and is required for the its full transcriptional activation (**Figure 5B**) (117, 118).

HDAC1 is a binding partner of AML1 that takes part in the forming of corepressor complex with nuclear receptor corepressor (N-CoR) and mammalian Sin3 (mSin3A and B) (119, 120). And ETO can bind to the central domain of N-CoR, generating the AML1-ETO/N-CoR/mSin3/HDAC1 complex, remodeling of chromatin structure and transcriptional suppression, dysregulating the normal hematopoiesis (26, 121). Additionally, the substrates of HDACs are not only histone but also non-histone proteins, such as oncogenes, tumor-suppressor genes, and chaperones. One of the presentative tumor suppressors is TP53. Specifically interacting with TP53, HDAC1 combined with the corepressor complex can mediated its deacetylation, and subsequent degradation. As a classical tumor suppressor, TP53 is crucial to the process of hematopoiesis. The alteration of TP53 is associated with the AML progress and therapy responsiveness, and generally predicts poor prognosis (9). Although its mutation frequency is relatively low in AML (less than 10% of *de novo* AML cases) compared to solid tumors (more than50% of cases), the function of TP53 in AML could not be ignored, as dysfunctional wild-type (WT) TP53 appears in various AML entities, implying a more attention to be paid (122).

HDAC2 are nearly identical to HDAC1, and usually work together in repressive complexes, such as nucleosome remodeling and deacetylase (NuRD), switch independent 3 (Sin3), and corepressor of RE1 silencing transcription factor (CoREST) complexes. Inhibition of HDAC1 and HDAC2 leads to down-regulation of RAD51, BRCA1, and CHK1, which are crucial for the DNA damage response (DDR) and subsequent DNA double-strand break and apoptosis in AML cell lines. And AML-1-ETO can bind with HDAC1, 2, and 3 to repress the AML1 target genes in t (8,21) AML (123). And HDAC6 deacetylates the chaperone Hsp90, eliciting the interaction with AML1-ETO protein, which can be dissociated by HDAC inhibitors that mediates the degradation of AML1-ETO protein.

HDAC11 may display a role in the immune system by regulating the immune cells. Antigen-presenting cells (APCs) plays critical role in T cell activation and tolerance, which is associated with HDAC11 (80). Up-regulation of HDAC11 can repress the expression of IL-10, and subsequently induce the APCs inflammation, which will prime naïve T cells and reactivate the response of tolerant CD4^+^ T cells. Meanwhile, down-regulation of HDAC11 in APCs promotes the expression of IL-10 and impairs the T cell response. Therefore, HDAC11 may act as a decider in the immune activation and tolerance, implying the substantial role of HDAC11 in the immunotherapy, involving in AML (80).

## HDACs in AML With CBFβ-MYH11

The inv (16) translocation emerges in 8-10% of AML patients, which is associated with M4Eo AML. It is produced by the chromosomal breakpoints within core binding factor beta (CBFB) gene on 16q22 and smooth muscle myosin heavy chain gene (MYH11) gene on 16p13, encoding corresponding proteins: CBFβ and smooth muscle myosin heavy chain (SMMHC). And the oncogenic gene CBFB-MYH11 and fusion protein CBFβ-SMMHC will subsequently generated and arrest the differentiation of hematopoietic cells. Similar to AML with AML1-ETO, the original disorder of AML with CBFB-MYH11 derives from the disruption of hematopoietic function performed by the core binding factor (CBF).

CBF, as a heterodimer, is composed of CBFα (DNA-binding subunit) and CBFβ (partner of CBFα) (124). CBFα subunit is encoded by CBFA2 that is known as RUXN1 or AML1 gene. CBFβ does not directly bind with DNA, but enhances the affinity of CBFα to DNA, stabilizing the CBFα-DNA complex. CBFβ-SMMHC fusion protein displays a higher affinity to AML1 binding than wild type CBFβ. Additionally, it contains an additional AML1-binding domain in SMMHC portion. Therefore, AML1 is preferential to bind with CBFβ-SMMHC, which competes the RUNX1-binding site with CBFβ, resulting in the blocks of AML1 function and enhancement of the SMMHC activity. The dysregulation of CBFβ acts an indirect factor disrupting the function of AML1, whose pivotal functions in hematopoiesis has been described in t (8,21) AML. Both CBFA2 and CBFB genes are indispensable for the development of normal hematopoiesis, deletion of either gene will disrupt the definitive hematopoietic stem cells. CBFβ-SMMHC protein interacts with the pivotal transcription factor AML1, sequestering the normal essential hematopoietic function of AML1. It acts as a transcriptional repressor, interacting with transcriptional inhibitors and HDACs, repressing the transcription of corresponding genes.

HDAC1 is a binding partner of AML1. And further research showed that HDAC1 can bind to CBFβ-SMMHC complex, which colocalizes with the promoters of AML1 and CBFβ-SMMHC. As a key cofactor, HDAC1 participates in the forming of AML1: CBFβ-SMMHC complex, which is essential for the transcriptional activity of related genes, involving in leukemic cell differentiation block and pro-proliferation (125). Additionally, pharmacologic inhibition of HDAC1 contributes to the suppression of leukemogenesis with CBFβ-SMMHC (126, 127). And *in vivo*, it can decrease the mouse leukemic burden, showing an effective role of HDAC1 targeting the CBFβ-SMMHC protein (30).

HDAC8, as another member of class I HDAC, has been demonstrated to interact with CBFβ-SMMHC protein. Besides, it can also reduce the acetylation of P53, which is bound to CBFβ-SMMHC protein, and subsequently promote the transformation of CBFβ-SMMHC-related leukemic stem cells. And inhibition of HDAC8 will induce the apoptosis in inv (16) AML (128, 129).

## HDACs in Acute Promyelocytic Leukemia With PML-RARA

The t (15,17) (q24;q21) translocation accounts for 10%-15% of acute promyelocytic leukemia (APL) issues. It is derived from the fusion of promyelocytic leukemia (PML) gene on 15q24 and retinoic acid receptor alpha (RARA) gene on 17q21, which is critical for the cellular transformation (130, 131).

PML gene is composed of nine exons that produces some alternative spliced transcripts variants, which share the N-terminal region, containing the RING-B-Box-Coiled-coil/tripartite motif (RBCC/TRIM) domain (132). Due to the alternative splicing, the isoforms of PML are different in central or C-terminal regions and the longest one is PML1, which harbors a nuclear export signal (NES) domain. In normal, PML mainly functions as a tumor suppressor. It can interact with over 170 proteins, most of which are mediated by the RBCC/TRIM domain leading to PML multimerization and organization or by other isoform-specific domains of PML. Conferred by these different binding interactions, PML is involved in proliferation and self-renewal of hematopoietic stem cells, epigenetic regulation in hematopoiesis, and p53-dependent/independent apoptosis and senescence (122). In addition, it is necessary for the formation of nuclear body (NB), which is associated with the protein release and sequestration, posttranscriptional modification, and promotion of nuclear issues (133).

RARA gene is consist of 10 exons producing two isoforms (RARA1 and RARA2) that are belonged to nuclear hormone receptor superfamily, acting as a nuclear transcriptional factor when retinoids are present, which is essential for the promyelocyte differentiation (130, 134). The RARA protein can interact with retinoid X receptor protein (RXRA), generating a heterodimer that acts as a transcription activator to bind with retinoic acid response elements (RARE). In the presence of ligand (all-trans retinoic acid (ATRA) or 9-cis retinoic acid), RARA binds to RXRA forming a heterodimer, which can interact with retinoic acid responsive elements (RARE). In the absence of ligand, RAR-RXR heterodimer recruits the transcriptional corepressors, such as HDACs, Sin3, SMRT, and N-CoR, keeping transcriptional repression, which can be dissociated when ligand emerges (135). In normal, RARA is ligand-dependent determining the transcriptional switch, which is critical for the differentiation of normal myeloid hematopoietic cells (134). In APL, the fusion protein PML-RARA alters the function of PML and RARA, disrupting the nuclear structure and blocking the cell differentiation. Additionally, PML-RARA provide leukemic cells with a survival and proliferative advantage, leading to the superiority accumulation of tumor cells in APL (130). Besides, through inducing the deacetylation of p53, PML-RARA fusion protein can directly suppress the activity of p53, conferring leukemic blasts to escape from p53-dependent cancer surveillance. And such phenomenon is realized by the recruitment of HDACs to PML-RARA complex, which can result in the deacetylation of p53 (136, 137).

PML–RARA recruits HDACs leading to RARs suppress the transcription of RA target genes, which displays a central role in the oncogenic transformation of APL (132). The aberrant recruitment of HDACs induced by PML–RARA contributes to the differentiation blocks and accumulation of APL blasts, because it inappropriately represses the RAR target genes. RA functioning as a therapeutic agent is based on the mechanism that RA can lead to the dissociation of PML–RAR/HDAC complex and degradation of such fusion protein (138, 139). Furthermore, ATRA resistance can be neutralized by HDAC inhibitors (140), which should have been paid more attention. Deregulated HDAC3 acts as a crucial role in the progress of acute promyelocytic leukemia (APL) with PML-RARα fusion protein. HDAC4 can interact with the PLZF-RARα fusion protein, mediating the differentiation arrest (141, 142).

## HDACs in AML With MLL-MLLT3

The t (9,11) AML presents in 3-5% of AML events, generated by the fusion of mixed lineage leukemia (MLL) gene on 11q23 and mixed lineage leukemia translocated to chromosome 3 (MLLT3) gene on 9p22, producing the fusion protein MLL-MLLT3 (143, 144).

MLL gene is made up of 14 exons, encoding the histone lysine methyltransferase whereby it is also called KMT2A, which harbors powerful transforming potential associated with neoplastic diseases assisted by specific partners, such as AF9 (MLLT3), AF4, and ENL (MLLT1) (145). It orchestrates various facets of cell development, including cell fate determine, stem cell maintenance, and embryogenesis. MLL protein contains multiple conserved domains with specific functions: 1) three AT hooks domains in the N-terminal of MLL mediating itself to bind with minor groove of DNA with AT-rich; 2) a transcriptional repressive domain that is composed of cysteine-rich CXXC DNMT (DNA methyltransferase1) homology region, which can bind to unmethylated CpG islands; 3) four plant homeodomain (PHD) fingers that mediate the protein-protein interactions; 4) a transactivation domain that is employed to interact with CBP/p300 complex; 5) SET [Su(var)3–9, enhancer of zeste, and trithorax] domain in C-terminal, serving as a histone H3 methyltransferase. Carrying along with such multiple domains, MLL can generate complexes with various partners, such as tumor suppressor Menin (multiple endocrine neoplasia), cell cycle regulator E2Fs, and HDACs (146, 147).

Overexpression of HDAC1, 2, and 3 is frequently found in leukemia (13). They can interact with MLL fusion protein leading to dysregulated chromatin remodeling, which could be neutralized by chidamide (148). Targeting MLL dysfunction by HDAC inhibitors such as vorinostat and panobinostat may counteract the aggressive resistance in MLL-fusion leukemia (149). And mocetinostat, a class I HDAC inhibitor, can inhibit the HOXA9 expression in AML with MLL-AF9 (147). Researchers have purified the stable MLL complex, where HDAC1 and HDAC2 were found. Additionally, they have also demonstrated that the repressive domain of MLL can specifically bind with HDAC1 and HDAC2, which can be partially released by HDAC inhibitor TSA but not RD1domain, which implies that additional cofactors are involved in the complex to fully perform the repressive function. And through binding to PHD fingers, Cyp33 can increase the affinity of MLL to HDAC1. Hypoacetylated histone in chromatin is frequently involved in transcriptionally repressive status (148, 150, 151).

## Concluding Remarks

HDACs serving as the pivotal epigenetic modifier of chromatin determine the chromatin accessibility to transcriptional factors, which is essential for specific gene transcription and oncogenic transformation. And the same to hematopoiesis, function of HDACs is indispensable, which determines the fate of hematopoietic cells, going through self-renewal, proliferation, differentiation, or apoptosis, terminating in various cell lineages (13, 14, 20). Thereby, dysregulation of HDACs inevitably leads to disruption of hematopoiesis (25, 91). It is necessary to concentrate on the investigation of HDACs functions.

The vital function of HDACs has long been acknowledged in the process of normal hematopoietic cell development and leukemogenesis, and numerous HDAC inhibitors have been applied in the treatment of various tumors but the mechanism of HDAC inhibitors serving in AML is elusive (20, 21). As the studies of HDACs function in AML increasing, we summarized the predominant importance in AML.

AML, with disrupted hematopoietic system, is usually hallmarked by oncogenic fusion proteins, majorly centralizing on AML1-ETO, CBFB-MYH11, PML-RARA, and MLL-AF9 (32, 33). HDAC inhibitors, the hyperacetylated agents, theoretically gear toward the alteration of the aberrant hypoacetylated status, providing a reasonable strategy against AML. They own the theoretical feasibility but practical hinderance, which provoked us to explore the precise function of HDAC, contributing to the utilization of HDAC inhibitors (152, 153). And mounting researches and reviews have demonstrated the roles of HDAC inhibitors in the treatment of AML. However, the function of HDACs in oncogenic molecules is rarely described (15, 26). Although the relative material is of shortage, it is meaningful to elucidate the potential function of HDACs in AML, focusing on the oncogenic fusion proteins that provides a directing target against specific types of AML.

Besides, HDACs display immunoregulatory properties in integral level, which overall regulates the progress of leukemogenesis through modulating the master elements of immune system such as PD-L1, CTLA-4, Treg, and cytotoxic T lymphocyte (CTL), and antigen-presenting cell (APC) (154–156). For instance, expression of HDAC10 is associated with the presentation of MHC class II molecules in antigen presentation cells (157, 158). Members of HDACs participate in the different stages of T cell development, including CD4^+^ T cell-mediated immunity (154, 159). That is to say, HDACs not only function with specific fusion proteins but also do regulate the entirety level of immune system which is involved in tumor microenvironment.

Attentions paid on HDACs usually focus on the HDAC inhibitors in the process of carcinogenesis, whereas the roles of HDACs have not got enough attention. It is necessary to harness the interaction between HDACs and leukemogenesis, which would precisely direct the investigation of novel HDAC inhibitors. Here, we summarized the current knowledge of HDACs functioning in leukemogenesis with oncogenic fusion proteins. They are closely associated with the suppression of oncogenic fusion genes, and can be blocked by HDAC inhibitors. However, pan-inhibitors presented various side effects and it can be improved by the specific HDAC inhibitors. And the searching of special targets is based on harnessing the traits of each HDAC member functioning in the epigenetic modification. The review summarized the functional properties of HDAC members, which may be useful for the exploration of specific HDAC inhibitors. Furthermore, HDACs is involved in the regulation of immune system, which may benefit to the investigation of novel agents or combinational drugs.

## Author Contributions

JZ drafted the manuscript. XG modified the manuscript. LY provided valuable advices, supervised, and approved the manuscript. All authors contributed to the article and approved the submitted version.

## Funding

This work was supported by grants National Natural Science Foundation of China (82030076, 82000161, 82070161, 81970151, 81670162, and 81870134), China Postdoctoral Science Foundation (2018M640824), Chinese National Major Project for New Drug Innovation (2019ZX09201002003), Shenzhen Science and Technology Foundation (JCYJ20190808163601776, JCYJ20200109113810154). Shenzhen Key Laboratory Foundation (ZDSYS20200811143757022). Sanming Project of Shenzhen (SZSM202111004).

## Conflict of Interest

The authors declare that the research was conducted in the absence of any commercial or financial relationships that could be construed as a potential conflict of interest.

## Publisher’s Note

All claims expressed in this article are solely those of the authors and do not necessarily represent those of their affiliated organizations, or those of the publisher, the editors and the reviewers. Any product that may be evaluated in this article, or claim that may be made by its manufacturer, is not guaranteed or endorsed by the publisher.
